# A hexadecylamide derivative of hyaluronan (HYMOVIS®) has superior beneficial effects on human osteoarthritic chondrocytes and synoviocytes than unmodified hyaluronan

**DOI:** 10.1186/1476-9255-10-26

**Published:** 2013-07-27

**Authors:** Margaret M Smith, Amy K Russell, Antonella Schiavinato, Christopher B Little

**Affiliations:** 1 Raymond Purves Bone and Joint Research Laboratories; Kolling Institute of Medical Research, Institute of Bone and Joint Research, University of Sydney at Royal North Shore Hospital, St Leonards, NSW, 2065, Australia; 2 Fidia Farmaceutici SpA, Abano Terme, Italy

**Keywords:** Osteoarthritis, Hyaluronan, Chondrocyte, Synovial fibroblasts

## Abstract

**Background:**

Intra-articular hyaluronan (HA) injection provides symptomatic benefit in the treatment of osteoarthritis (OA). Previously we found superior beneficial effects in a large animal OA model of a hexadecylamide derivative compared with unmodified HA of the same initial molecular weight. The current study sought to define possible molecular mechanisms whereby this enhanced relief of symptoms was occurring.

**Methods:**

Chondrocytes and synovial fibroblasts were isolated from tissues of patients undergoing arthroplasty for knee OA. Monolayer cultures of cells were treated with 0, 0.5, 1.0 or 1.5 mg/mL of unmodified HA (500–730 kDa) or a hexadecylamide derivative of HA of the same initial molecular weight (HYADD4®-G; HYMOVIS®) simultaneously or 1 hour before incubation with interleukin (IL)-1beta (2 ng/mL). Cultures were terminated 15 or 30 minutes later (chondrocytes and synovial fibroblasts, respectively) for quantitation of phosphorylated-(p)-JNK, p-NFkappaB, p-p38, or at 24 hours for quantitation of gene expression (*MMP1* &*13, ADAMTS4* &*5*, *TIMP1* &*3*, *CD44*, *COL1A1* &*2A1*, *ACAN*, *PTGS2*, *IL6*, *TNF)* and matrix metalloproteinase (MMP)-13 activity.

**Results:**

The hexadecylamide derivative of HA had significantly better amelioration of IL-1beta-induced gene expression of key matrix degrading enzymes (*MMP1, MMP13, ADAMTS5*), and inflammatory mediators (*IL6, PTGS2*) by human OA chondrocytes and synovial fibroblasts. Pre-incubation of cells with the derivatized HA for 1 hour prior to IL-1beta exposure significantly augmented the inhibition of *MMP1, MMP13, ADAMTS4* and *IL6* expression by chondrocytes. The reduction in *MMP13* mRNA by the amide derivative of HA was mirrored in reduced MMP-13 protein and enzyme activity in IL-1beta-stimulated chondrocytes. This was associated in part with a greater inhibition of phosphorylation of the cell signalling molecules JNK, p38 and NF-kappaB.

**Conclusions:**

The present studies have demonstrated several potential key mechanisms whereby the intra-articular injection of a hexadecylamide derivative of HA may be acting in joints with OA.

## Background

Osteoarthritis (OA) is characterized by joint pain and stiffness with accompanying disability and loss of quality of life. All joint tissues, including capsule, synovium, intra-articular ligaments and menisci, bone and cartilage are affected in OA. The articular cartilage within affected joints is eventually degraded and lost, thus ceasing to function as a frictionless bearing surface. While it is the ultimate loss of cartilage that often necessitates the need for joint replacement, it is subchondral bone lesions and synovial/joint capsule inflammation and fibrosis that appear to be primarily responsible for OA pain [1]. Current treatments for OA are limited and are either pain-relieving or involve joint replacement at end-stage disease. There are no treatments available that have been unequivocally demonstrated to exhibit structural modification to restore function.

Hyaluronan (HA), a macromolecular glycosaminoglycan, is a major component of both synovial fluid and articular cartilage. Treatment of OA with intra-articular HA is a current alternative treatment to NSAIDs or other palliative pain medications and a number of clinical trials have been performed with different HA products. Recent meta-analyses have concluded that HA is of a longer-term symptomatic benefit than other treatments, with a clinically relevant effect size equivalent or greater than other analgesics [2,3]. Despite this evidence, the mechanism(s) by which HA alleviates joint pain remains unclear. Intra-articular HA therapy can lead to modulation of nociceptors [4], reduction of synovial/capsular fibrosis [5], increased synthesis of higher molecular weight endogenous HA by the resident synoviocytes [5,6], and reduction in inflammatory mediators including prostaglandin-E2, IL-1 and IL-6 [7-9]. There is significant evidence that intra-articular HA injection reduces the rate of cartilage degeneration in animal models of OA [10,11] (and reviewed in [4,12]) but whether the same is true in humans is unclear. Amelioration of cartilage breakdown by HA is thought to occur through enhancement of chondrocyte aggrecan synthesis [13,14], reduction of matrix degrading enzyme (matrix metalloproteinases (MMPs) [15] and disintegrin and metalloproteinase with thrombospondin motifs (ADAMTS) [16]), and modification of inflammatory cytokine expression and activity [4]. The latter also being implicated in symptom modification. The effectiveness of intra-articular HA on both symptomatic and structural modification in pre-clinical OA models, appears to be dependent, at least in part, on its MW [17,18].

Previously we found superior beneficial effects of intra-articular injection of a hexadecylamide derivative of HA (HYADD4®-G; HYMOVIS®) compared with unmodified 500–730 kDa HA, on gait analysis, stimulation of synoviocyte high MW HA synthesis, and reduction in synovial hyperplasia in model of established OA in sheep [5,19]. The current study investigated the potential mechanisms whereby HA might be acting using human joint tissue cells, and whether these differed between the amide derivative and unmodified HA preparations. We found a significantly greater effect of the hexadecylamide derivative of HA in ameliorating IL-1-induced expression and/or activity of key matrix degrading enzymes (*MMP1, MMP13, ADAMTS5*), and inflammatory mediators (*IL6*, *PTGS2*) by chondrocytes and synovial fibroblasts. This was associated in part with a greater inhibition of cell signalling molecule (JNK, p38 and NFκB) phosphorylation in chondrocytes, together suggesting mechanisms whereby the hexadecylamide derivative of HA may be beneficial.

## Methods

### Ethics

Ethics approval was obtained from North Shore Central Coast Area Health and Ramsey Health to collect discarded human joint tissue from OA patients undergoing total or partial knee replacement surgery at Royal North Shore Hospital and North Shore Private Hospital respectively (Northern Sydney Central Coast Health 0902-034 M, NSW Health). The protocol required written patient consent and was managed through the Department of Orthopaedics and Traumatic Surgery at Royal North Shore Hospital.

### Patient demographics

Tissues (either synovium or articular cartilage) were harvested from a total of 19 patients (9 male, 10 female; 7 HSF and 12 HAC) with a mean age of 70 years (range 55 – 86). All patients except one were undergoing either a unilateral or bilateral total knee replacement; the youngest patient had a partial replacement.

### Cell isolation and culture

Human articular chondrocytes (HAC) were isolated from articular cartilage and synovial fibroblasts (HSF) from synovium by proteolytic digestion using established methods [6,20]. All HSF were cryopreserved before use; all HAC were used immediately after isolation at the primary passage. Cells were cultured in monolayer in DMEM (HSF) or DMEM/F12 (HAC) media supplemented with 10% (v/v) foetal bovine serum (FBS), 2 mM glutamine to confluence after seeding at 300,000 cells/well in 6-well plates. Primary chondrocytes were used immediately after isolation from tissue digests to avoid any loss of matrix expression due to dedifferentiation [21] and fibroblasts were used at the 6th to 10th passage. Cells from at least 5 different patients in triplicate wells per condition were used for each experiment.

After reaching confluence, cells were washed with serum free media and then cultured for 24 hours in fresh serum-free media (HAC) or with 1% (v/v) FBS (HSF) for acclimatization. Fresh media containing unmodified or the hexadecylamide derivative of HA (0, 0.5, 1, and 1.5 mg/mL) was added and cells cultured with either simultaneous addition or delayed (after one hour pre-incubation with HA) addition of recombinant IL-1β (2 ng/mL). Cultures were terminated at different time points after the addition of IL-1β depending on the outcome measure: 1) 24 hours for gene expression analysis and enzyme activity or 2) 15 minutes (HAC) or 30 mins (HSF) for phosphoprotein Bio-plex analysis (see below).

In HSF from 1 patient and HAC from 2 patients, CD44 antibody (R&D systems BBA10; 5 or 10 μg/mL) or IgG isotype control antibody at the same concentration (R&D Systems MAB003) were added to the cultures 1 or 3 hours before the addition of the hexadecylamide derivative of HA (HYADD4®-G; HYMOVIS® at 0.5 mg/ml), ± IL-1β (2 ng/mL) one hour later, as previously described [22]. These cultures (n = 6 replicates for each treatment in each patient) were terminated at 24 hours for gene expression analysis.

### Preparation of HA media solutions

Unmodified HA (500–730 kDa 10 mg/ml in 2 ml sterile syringe) and a hexadecylamide derivative of HA of the same initial molecular weight (HYADD®4-G; HYMOVIS®; 8 mg/ml in 3 ml sterile syringe) of the same purity were supplied by Fidia Farmaceutici SpA. For treatment of each cell type from each patient, 3 mg/ml solutions of unmodified or the hexadecylamide derivative of HA in appropriate 1× culture media (6 ml HYMOVIS® + 2 ml sterile H_2_O or 4.8 ml unmodified HA + 3.2 ml H_2_O; added to 8 ml 2× culture media) were freshly prepared the day before and very gently rotated overnight at 4°C to ensure a homogeneous solution. This procedure is necessary due to the high viscosity of these solutions. These 3 mg/ml stocks of HA were then appropriately diluted in media to the physiologically relevant concentrations of 1.5, 1.0 and 0.5 mg/ml [23].

### RNA extraction, reverse transcription and real time PCR

At the termination of culture, cells for RNA extraction were rinsed twice gently with phosphate buffer saline pH 7.2 and then removed from the wells using 0.75 mL TRIzol®. Total RNA was extracted using Qiagen RNeasy minikits with an on-column DNase digestion. RNA was quantitated using a Nanodrop® spectrophotometer. RNA quality was assessed by Nanodrop®, MultiNA microchip electrophoresis and RT-PCR using GAPDH primers to test for the presence of genomic DNA. A uniform amount of RNA of sufficient quality was reverse transcribed using a Qiagen Omniscript kit with added random decapentamers and RNase inhibitor (Bioline). Real time PCR was performed using validated human specific primers for *MMP1*, *MMP13*, *ADAMTS4*, *ADAMTS5*, *TIMP1*, *TIMP3*, *CD44*, *PGTS2*, *IL6*, *TNF*, *FN1*, *ITGA5*, *GAPDH*, *ACAN*, *COL2A1* and *COL1A1* (details in Additional file 1: Table S1) and internal standards (random mix of HSF/HAC cDNA) using a Rotor-gene 6000 (Qiagen) as previously described [24]. The Rotor-gene 6000 Series software (version 1.7) generated relative fluorescent units (RFU) for sample cycle thresholds based on calculated, optimized, baseline- and slope-corrected thresholds generated by tissue-matched cDNA standards. Results were corrected for total RNA as previously recommended [25,26] to avoid bias from regulation of house-keeping genes typically observed in mechanically-loaded tissues [27], and expressed as fold change from control (no added HA) cultures of the same patient.

### MMP-13 activity and protein levels

Media was harvested and analysed for MMP-13 activity using a commercially available fluorogenic assay (SensoLyte Plus™ 520 MMP-13 Assay Kit; Anaspec, San Jose, CA, USA). MMP-13 activity was measured with and without activation by aminophenylmercuric acetate (APMA). Fluorescence was detected every 30 minutes for 4 hours and MMP-13 activity reported as change in relative fluorescent units (RFU)/hour. For Western blot analysis, media was digested with *Streptomyces* hyaluronidase (20 turbidity reducing units/mL at 60°C for 3 hours). Proteins were then precipitated with 5 volumes of ethanol at 4°C for 16 hours, redissolved in reducing SDS-PAGE buffer, boiled for 5 minutes and separated on 10% SDS-PAGE gels (Invitrogen). Separated proteins were transferred to nitrocellulose membranes and MMP-13 protein detected using a monoclonal antibody (Abcam 39012) [28,29].

### Quantitation of cellular phosphoproteins

Cells for protein extraction were lysed according to the supplied Bio-plex® protocol. Total protein in all samples was measured using the bicinchoninic acid assay [30]. Phosphorylated p38 MAPK, JNK and NFκB and total JNK and p38 MAPK were measured using commercial kits (Bio-Rad Laboratories) and a Bio-Plex® multiplex analyser. A kit for quantifying total NFκB on the Bio-Plex® multiplex analyser was not available at the time these experiments were conducted.

### Statistical analyses

Differences in real time PCR results between IL-1β or HA treatments in HAC or HSF cell cultures were compared using a paired rank sum nonparametric test, as data was not normally distributed. The fold change from no treatment (no HA and no IL-1) within each cell type from each patient was used in order to correct for differences in baseline expression between the patients. Gene expression results are presented graphically as a mean log fold change from the control cultures (no IL-1β; no HA). Bio-plex phosphoprotein and MMP-13 activity data was analyzed using paired Students t tests between outcomes in the presence and absence of HA, and unpaired tests between with and without preincubation. All analyses were performed using Stata 11.2.

## Results

### Cell viability

Cells remained viable throughout all experiments, with preliminary trials revealing no change in cell numbers with any treatment (data not shown) as previously published [22]. Some HSF detached from the culture well surface in the absence of FBS and this phenomenon was exacerbated in the presence of HA. To prevent this occurring, all HSF studies were carried out in the presence of 1%(v/v) FBS.

### Effect of IL-1β, unmodified HA and the amide derivative of HA on gene expression by chondrocytes

**Figure 1:**
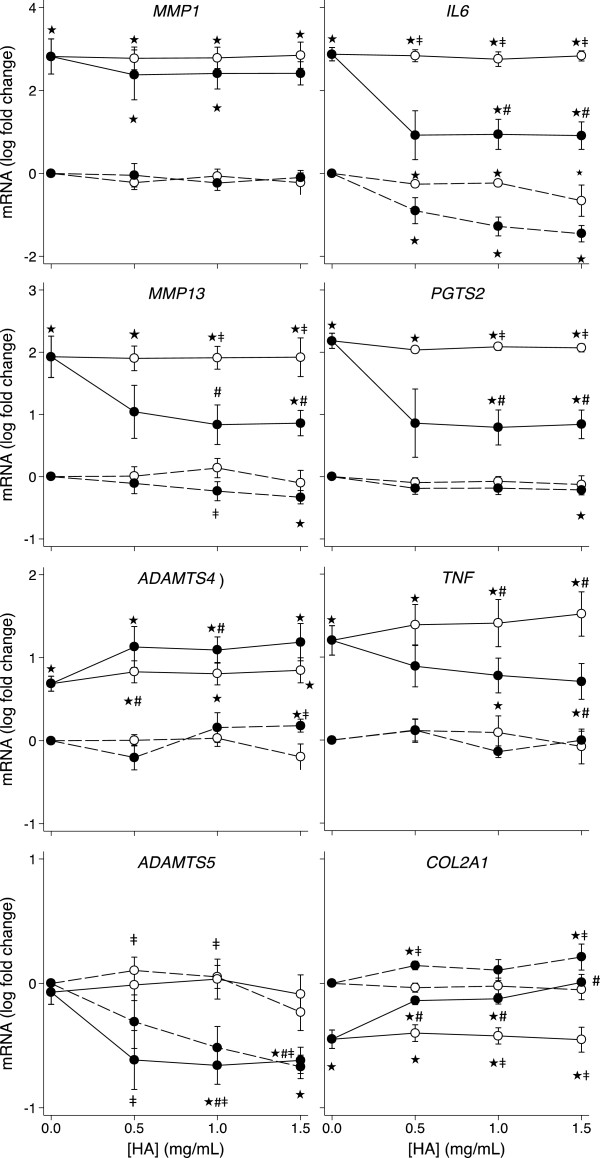
**HA dose response of HAC gene expression.** Dose response of unmodified HA (white markers) and the amide derivative of HA (black markers) on HAC expression of the indicated genes in the presence (solid line) and absence (dashed line) of IL-1β (2 ng/mL). Values are mean log fold-change from control (no IL-1 no HA; at zero) from six separate patients. Note different scales within each row. *P* < 0.05* different from no IL-1, no HA control; # different from IL-1, no HA control; ‡ different between the amide derivative and unmodified HA at the same concentration.

The effects of IL-1 ± simultaneous addition of either HA preparation on human OA chondrocytes are summarised in Figure 1 (and Additional file 2: Figure S1). In the absence of IL-1 stimulation, effects were seen predominantly with the derivatized compared with unmodified HA, with the former inducing a 10 fold reduction in *IL6* (*P* = 0.028), a 4 fold decrease in *ADAMTS5* (*P* = 0.028), and also increasing *COL2A1* (*P* = 0.028) expression by an average of 60% at one or more doses. At the highest dose (1.5 mg/ml), the amide derivative but not unmodified HA, also reduced by an average of 45% (range 4 – 75%) the expression of *MMP13* and *PGTS2* (*P* < 0.05). In human OA chondrocytes IL-1β significantly (*P* < 0.05) increased expression of *MMP1* (9100 fold), *MMP13* (660 fold), *ADAMTS4* (5.5 fold), *TNF* (21 fold), *IL6* (1020 fold), *PGTS2* (180 fold), *TIMP3* (76%) and significantly decreased expression of *COL2A1* (2.6 fold), and *ACAN* (2 fold). There was no significant effect of IL-1β on chondrocyte expression of *ADAMTS5*, *CD44*, *TIMP1*, *FN1*, *ITGA5* or *GAPDH*. As noted in the absence of IL-1, the derivatized HA preparation also had a more profound effect on chondrocyte gene expression than unmodified HA in IL-1-treated cultures. In addition to significantly reducing *IL6* (24 fold), *ADAMTS5* (3.5 fold) and increasing *COL2A1* (2 fold) in IL-1-stimulated cultures, the HA hexadecylamide derivative significantly ameliorated the IL-1β-induced changes in HAC expression of *MMP13* (45%), *PGTS2* (11 fold), *TNF* (2.4 fold), and *ACAN* (50%) at one or more of the concentrations tested. Unmodified HA at 1.0 and 1.5 mg/ml significantly increased further the IL-1β-augmented expression of *TNF* and *TIMP3* (*P* = 0.043) but no other genes. The hexadecylamide derivative of HA increased further the IL-1β-augmented expression of *ADAMTS4* (*P* = 0.043 at 1.0 mg/ml) but no other genes.

### Effect of IL-1β, unmodified HA and the amide derivative of HA on gene expression by synovial fibroblasts

**Figure 2:**
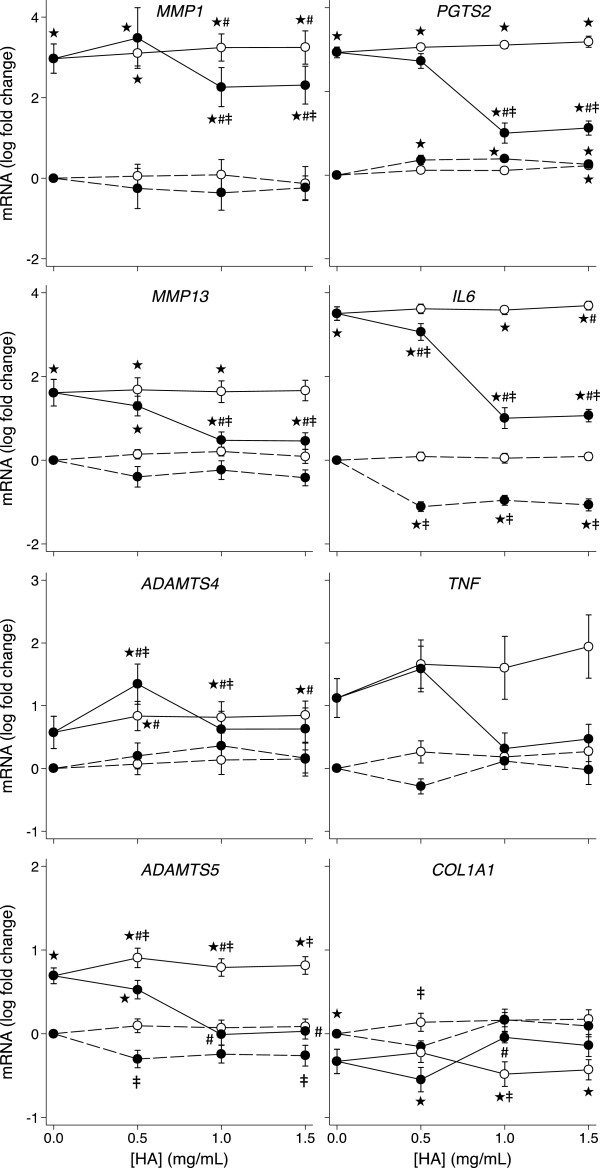
**HA dose response of HSF gene expression.** Dose response of unmodified HA (white markers) and the amide derivative of HA (black markers) on HSF expression of the indicated genes in the presence (solid line) and absence (dashed line) of IL-1β (2 ng/mL). Values are mean log fold-change from control (no IL-1 no HA; at zero) from six separate patients. Note different scales between rows. *P* < 0.05* different from no IL-1, no HA control; # different from IL-1, no HA control; ‡ different between the amide derivative and unmodified HA at the same concentration.

The effects of IL-1β and the HA preparations on OA human synovial fibroblasts (HSF) are summarized in Figure 2 (and Additional file 3: Figure S2). As in HAC, effects on HSF in the absence of IL-1 stimulation were seen predominantly with the derivatized HA compared with unmodified HA. The hexadecylamide derivative of HA, but not unmodified HA, at one or more concentrations decreased the expression of *TIMP1* (40%), *TIMP3* (2.0 fold), *IL6* (9 fold) and *CD44* (1.7 fold) in the absence of IL-1β (*P* < 0.05). Unmodified HA at 1.5 mg/ml and the amide derivative of HA at all tested concentrations increased the expression of *PGTS2* up to 5.6 fold in the absence of IL-1β (*P* = 0.043). IL-1β induced significantly increased expression of *MMP1* (3200 fold), *MMP13* (200 fold), *ADAMTS5* (8 fold), *IL6* (4040 fold), *PGTS2* (1090 fold) and significantly decreased expression of *COL1A1* (40%) in the synovial fibroblast cultures (*P* < 0.05). There was no significant effect of IL-1β on synovial fibroblast expression of *ADAMTS4*, *CD44*, *TIMP1*, *TIMP3*, *TNF*, *FN1*, *ITGA5* or *GAPDH*. The derivatized HA preparation again had a more profound beneficial effect on HSF gene expression than unmodified HA in IL-1-treated cultures. Derivatized HA partially but significantly ameliorated the IL-1β-induced changes in HSF expression of *MMP1* (by 70%), *MMP13* (40 fold lower), *PGTS2* (5 fold lower), *IL6* (24 fold lower) and completely reversed the IL-1β-induced changes in HSF expression of *ADAMTS5, TIMP1, COL1A1* and *CD44* (all *P* < 0.05). Unmodified HA increased further the IL-1β-augmented expression of *MMP1* at 1.0 and 1.5 mg/ml (34%), *ADAMTS5* at 0.5 and 1.0 mg/ml (50%) and IL6 at 1.5 mg/ml (40%) (*P* = 0.043) in the HSF cultures. Unmodified HA at all concentrations and the amide derivative of HA at 0.5 mg/ml significantly increased (60% - 7 fold) HSF *ADAMTS4* expression in the presence of IL-1β (*P* < 0.05).

### Effect of pre-incubation with the amide derivative of HA on IL-1β-induced gene expression changes

**Figure 3:**
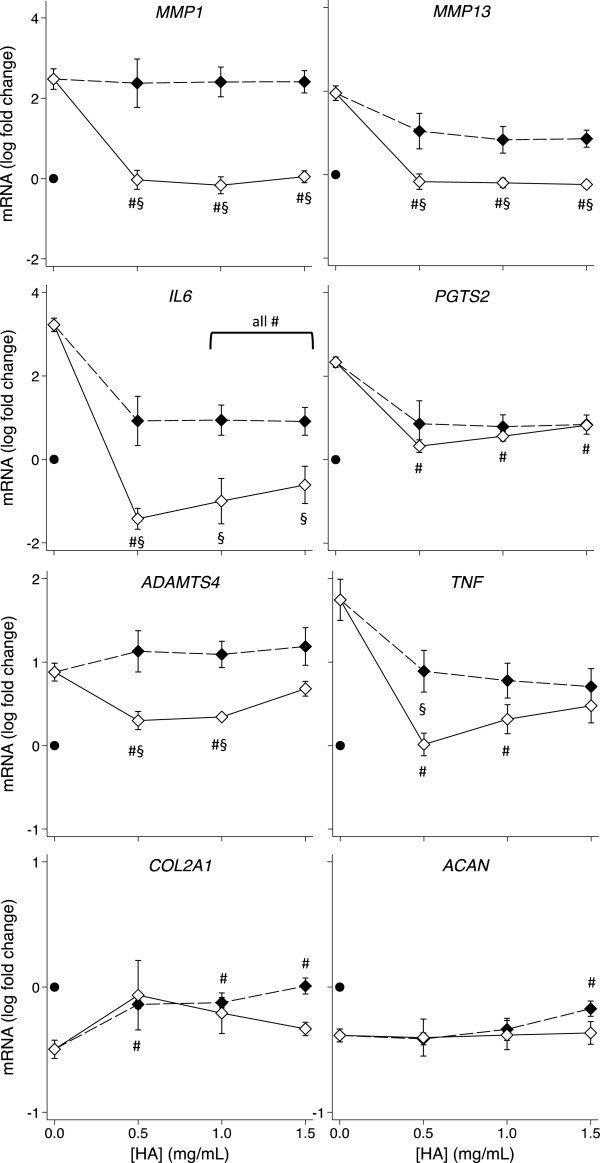
**Effect of pre-incubation on the HA dose response of HAC gene expression.** Dose response on expression of the indicated genes of the hexadecylamide derivative of HA added simultaneously with (black markers, dashed line) or 1 hour before (white markers, solid line) the addition of IL-1β (2 ng/mL) in cultures of HAC. *P* < 0.05 for differences from cultures with IL-1β alone (no added HA; #) or differences +/− pre-incubation (§). Values are mean log fold-change from control (no IL-1 no HA; black dot) from five separate patients.

**Figure 4:**
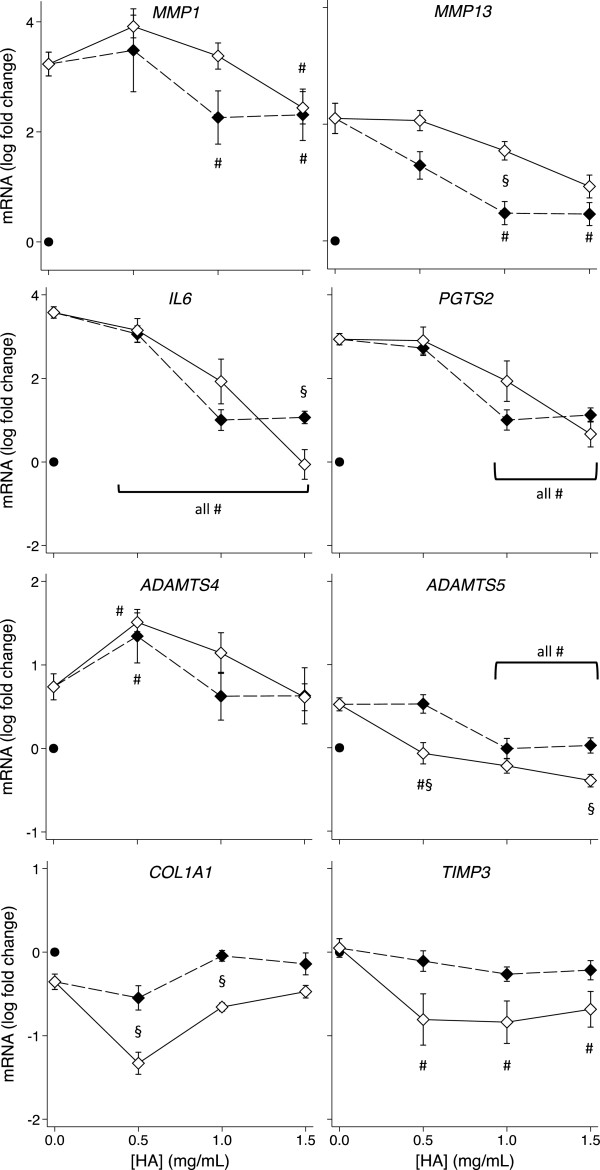
**Effect of pre-incubation on the HA dose response of HSF gene expression.** Dose response on expression of the indicated genes of the hexadecylamide derivative of HA added simultaneously with (black markers, dashed line) or 1 hour before (white markers, solid line) the addition of IL-1β (2 ng/mL) in cultures of HSF. *P* < 0.05 for differences from cultures with IL-1β alone (no added HA; #) or differences +/− pre-incubation (§). Values are mean log fold-change from control (no IL-1 no HA; black dot) from five separate patients.

The above data indicated that the derivatized HA had a pronounced effect on OA chondrocyte and synovial fibroblast gene expression when added simultaneously with IL-1. We investigated whether the same or different effects may be observed if cells were pre-incubated with HYMOVIS® prior to IL-1 exposure. Figures 3 and 4 (also Additional file 4: Figures S3 and Additional file 5: Figure S4) compare the gene expression by HAC and HSF respectively with and without one hour of pre-incubation with the hexadecylamide derivative of HA before the addition of IL-1β. Pre-incubation significantly enhanced the amelioration of the IL-1β-induced changes in HAC expression of *MMP13*, *IL6* and *TNF* (*P* < 0.05) but not *PGTS2* or *ADAMTS5*. IL-1β-induced *MMP1* expression by HAC was completely reversed by all concentrations of the hexadecylamide derivative of HA and *ADAMTS4* expression was normalized by 0.5 and 1.0 mg/mL derivatized HA after pre-incubation, whereas simultaneous addition of the hexadecylamide derivative of HA with IL-1β had no effect on these genes (Figure 3). In contrast to the enhanced effects on HAC, pre-incubation with the derivatized HA had minimal beneficial effects compared with simultaneous addition in HSF, significantly decreasing *COL1A1* at the two lower doses and *IL6* and *ADAMTS5* at the highest dose (Figure 4).

### Effect of blocking CD44 on effects of pre-incubation with the amide derivative of HA

Addition of anti-CD44 at 5 μg/mL for 1 hour partially ameliorated (~50%) the inhibition by the derivatized HA of IL-1-induced *IL6* and *TNF* expression in HAC from one patient. However this was not statistically significant and there was no change in the effect of the derivatized HA on other genes (*PTGS2, MMP1, MMP13*, *ADAMTS4*). There was no change in the effect of the derivatized HA on gene expression in HAC from another patient when pre-treated for 3 hours with 10 μg/mL anti-CD44. In HSF there was a significant reversal by anti-CD44 of the effect of the hexadecylamide derivative of HA on expression of *MMP13* (P < 0.001) and *ADAMTS4* (P = 0.025) in the absence of IL-1 stimulation (data not shown). However there was no modulation by anti-CD44 on the effect of HA on HSF gene expression in the presence of IL-1.

### Effect of HA and its amide derivative on MMP-13 protein and activity

**Figure 5:**
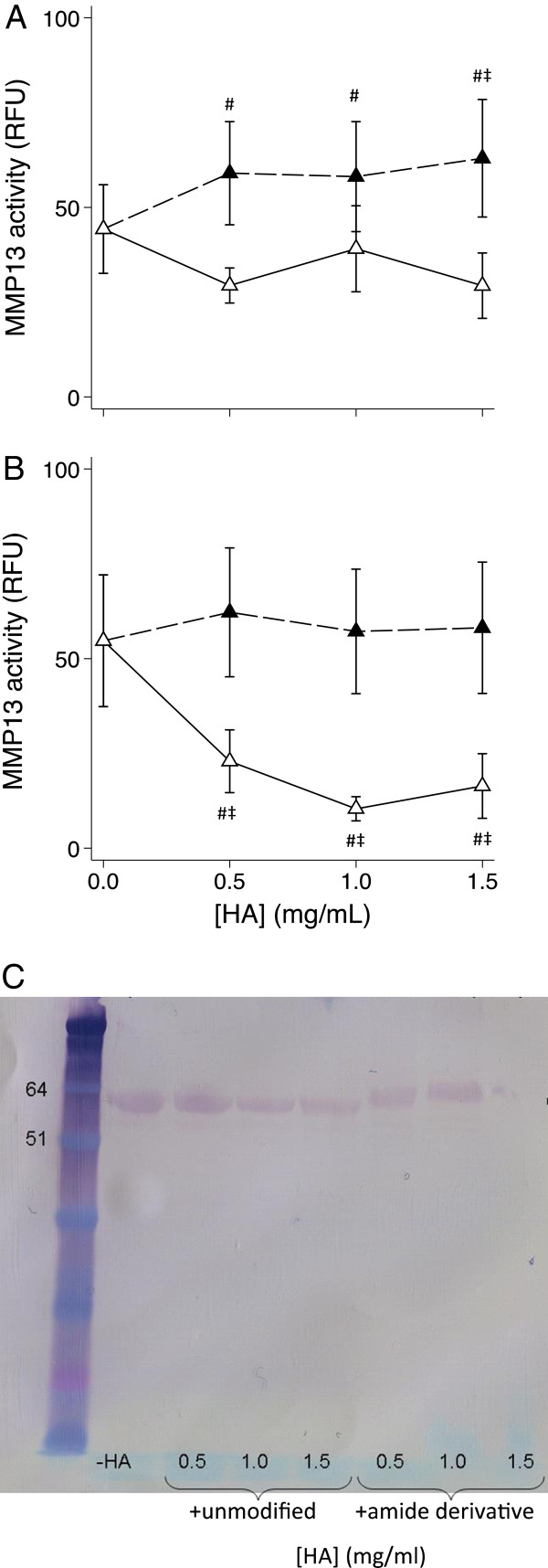
**HA dose response on MMP-13 activity and protein.** Dose response of the effect of unmodified HA (black) or the hexadecylamide derivative of HA (white) on APMA-activated MMP13 activity in the culture media after exposure to IL-1β (2 ng/mL) for 24 hours in HSF **(A)** or HAC **(B)**. *P* < 0.05 # different from IL-1, no HA control; ‡ different between the amide derivative and unmodified HA at the same concentration. **(C)** Western blot of MMP-13 protein in pooled media from HAC incubated with and without unmodified or the hexadecylamide derivative of HA at the indicated concentrations. Migration of pre-stained molecular mass markers (kDa) is shown on the left.

No MMP-13 activity was detected in conditioned media in the absence of APMA (data not shown), so all subsequent results relate to APMA-activated media. Unmodified HA at all concentrations tested increased MMP-13 activity by 31 – 42% (*P* < 0.015) in media from HSF (n = 6, Figure 5A) but no significant effect on this enzyme in media from HAC (n = 6, Figure 5B) was found, perhaps due to greater variability between cell cultures from different patients. The hexadecylamide derivative of HA did not affect HSF MMP-13 activity but reduced HAC activity by 62% (*P* = 0.045), 79% (*P* = 0.006) and 74% (*P* = 0.006) at 0.5, 1.0 and 1.5 mg/ml respectively (Figure 5B).

Western blotting for MMP-13 in HSF conditioned media was unsuccessful due to the presence of FBS in these cultures (data not shown). MMP-13 protein was immuno-detected in a Western blot of pooled media from HAC cultured in the presence and absence of unmodified and the hexadecylamide derivative of HA (Figure 5C). The migration position in the absence and presence of unmodified HA was consistent with pro-MMP-13 (~60 kDa). In the presence of derivatized HA the relative molecular mass of the MMP-13 band was somewhat increased (~65 kDa), and there was a notable reduction in band intensity in the media of cells cultured with the highest dose of derivatized HA.

### Effect of the hexadecylamide derivative of HA with and without pre-incubation on nuclear phosphoproteins

**Figure 6:**
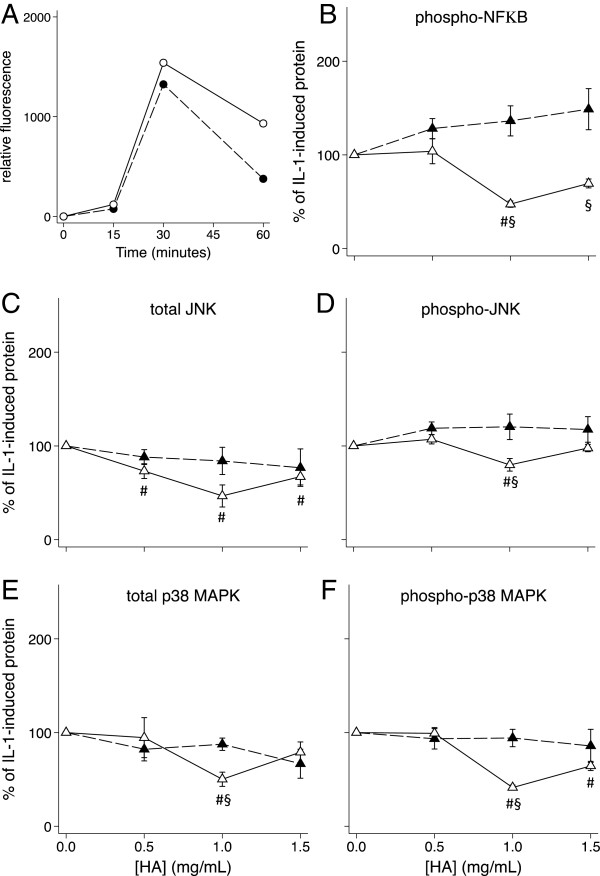
**Effect of HA on HSF phosphoproteins. A)** Time course (minutes) of the appearance of phosphorylated p38 (black markers, dashed line) and JNK (white markers, solid line) in HSF after the addition of IL-1β. **B)** – **F)** Dose response of the effect on levels of the indicated signalling proteins of the hexadecylamide derivative of HA added one hour before (white markers, solid line) or simultaneously with (black markers, dashed line) of IL-1β (2 ng/mL) to cultures of HSF. Cultures were harvested after 30 minutes with IL-1β corresponding to peak levels **(A)**. *P* < 0.05 for differences from cultures with IL-1β alone (no added HA; #) or differences +/− pre-incubation (§). Values are mean % change from IL-1 alone in cells from five separate patients.

**Figure 7:**
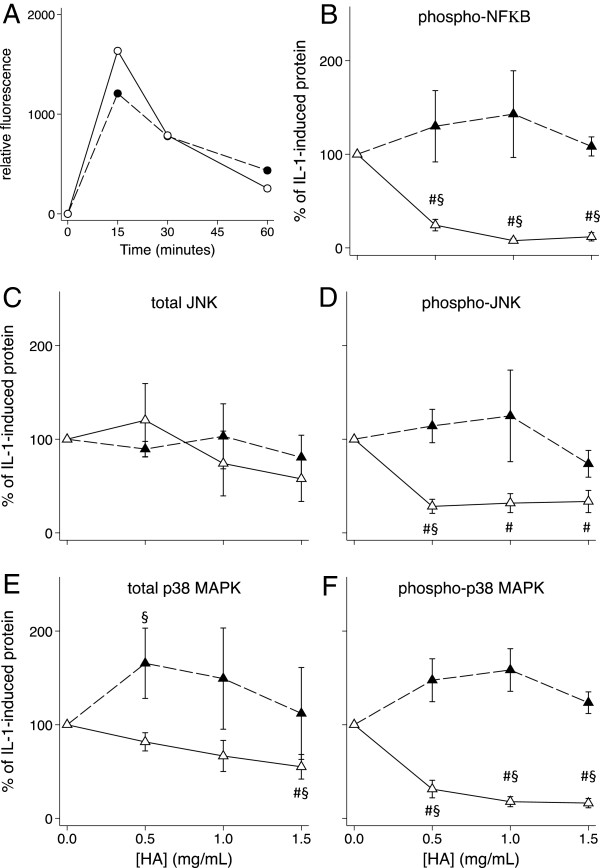
**Effect of HA on HAC phosphoproteins. A)** Time course (minutes) of the appearance of phosphorylated p38 (black) and JNK (white) in HAC after the addition of IL-1β. **B)** – **F)** Dose response of the effect on levels of the indicated signalling proteins of the hexadecylamide derivative of HA added one hour before (white markers, solid line) or simultaneously with (black markers, dashed line) of IL-1β (2 ng/mL) to cultures of HAC. Cultures were harvested after 15 minutes with IL-1β corresponding to peak levels **(A)**. *P* < 0.05 for differences from cultures with IL-1β alone (no added HA; #) or differences +/− pre-incubation (§). Values are mean% change from IL-1 alone in cells from five separate patients.

Phosphorylation was detectable in culture for 60 minutes after addition of IL-1β. The HSF and HAC phosphoprotein levels peaked at 15 and 30 minutes respectively after IL-1β addition (Figures 6A and 7A). These two timepoints were subsequently used to measure the effects of the hexadecylamide derivative of HA on levels of total and phosphorylated phosphoproteins. Simultaneous addition of the hexadecylamide derivative of HA and IL-1β to either HSF or HAC cultures did not significantly alter levels of total JNK or p38 MAPK or phosphorylated JNK, p38 MAPK or NFκB (Figures 6 and 7B-F). However, IL-1 stimulated levels in HSF of total (*P* = 0.009; Figure 6E) and phosphorylated (*P* = 0.001; Figure 6F) p38 MAPK decreased by ~50% by one hour preincubation with 1.0 mg/ml derivatized HA. Phosphorylated NFκB, total JNK and phosphorylated JNK were also significantly reduced by 50% (*P* = 0.001), 60% (*P* = 0.024) and 20% (*P* = 0.024) respectively by this same treatment (Figure 6B-D). In HSF cultures, the changes in phosphorylated protein levels induced by pre-incubation with the hexadecylamide derivative of HA closely mirrored changes in the total phosphoprotein levels, suggesting the major effect in this cell type was on total phosphoprotein. Conversely in HAC, pre-incubation with all concentrations of the hexadecylamide derivative of HA significantly decreased the levels of phosphorylated p38 MAPK to less than 30% (*P* = 0.001; Figure 7F) without significantly changing total p38 MAPK levels (Figure 7E). Similarly, pre-incubation with all concentrations of the hexadecylamide derivative of HA significantly decreased the levels of phosphorylated JNK to 30 – 35% (*P* = 0.001; Figure 7D) without significantly altering total JNK levels in HAC (Figure 7C). Phosphorylated NFκB in HAC was reduced to 22%, 10% and 6% by pre-incubation of HAC with 0.5, 1.0 and 1.5 mg/mL derivatized HA respectively (all *P* = 0.001; Figure 7B).

## Discussion

Intra-articular injection of HA is a clinically beneficial therapy for OA with meta-analysis generally demonstrating prolonged symptomatic pain relief [2,3]. The mechanisms for this clinical effect remain unclear given the apparently short half-life of exogenous HA in the joint, and that long-term modulation of OA disease progression has not been confirmed in human studies. There is evidence from animal models to suggest that the beneficial effects of HA may be at least in part dependent on its molecular weight and potentially its clearance from the joint [17,18]. We previously demonstrated that a novel hexadecylamide derivative of HA (HYADD4®-G; HYMOVIS®) was superior to unmodified HA of the same initial molecular weight, in terms of reducing lameness and synovial hyperplasia when administered in established OA in sheep [5,19], but the mechanisms behind this have not been established.

In the present study we utilized cultures of OA HAC and HSF derived from total knee replacement patients with and without added IL-1β, to investigate the molecular mechanisms whereby the hexadecylamide derivative of HA may have positive effects on cartilage-degrading activities. HAC and HSF from OA joints maintain a pathological phenotype in culture but enhanced pro-catabolic and pro-inflammatory responses can still be induced by cytokine stimulation. Although often considered a “non-inflammatory” joint disease, it is increasingly being recognized that cytokines, particularly IL-1β, and tumor necrosis factor-α (TNFα) may play an important role in both the progressive degradation of the cartilage and the disease symptoms (reviewed in [31,32]). These cytokines can directly inhibit expression of cartilage extracellular matrix components, stimulate expression of MMPs and ADAMTS that degrade cartilage, and stimulate the production of other pro-inflammatory molecules such as IL-6 and nitric oxide [31-34]. We did not see a stimulation of ADAMTS5 in these human cells as has been seen previously in lapine chondrocytes [35], perhaps due to a species difference. However the *in vitro* effects of IL-1 on ADAMTS5, as with many genes, can vary with cytokine concentration and incubation time, which may also explain the differences observed. Interestingly, blockade of TNFα reduces joint inflammation while inhibition of IL-1β protects against cartilage degradation in mouse arthritis models, suggesting the latter may be an attractive molecular target in the development of disease-modifying therapies [36]. Studying the effect of therapeutic agents on HAC and HSF ± IL-1β *in vitro* may, therefore, provide insights into potential regulatory pathways and this approach has been widely used to evaluate other HA formulations [6,16,37-44].

We have demonstrated that the hexadecylamide derivative of HA had a profound effect in reducing expression of key proteolytic enzymes implicated in cartilage degradation in OA (*MMP1, MMP13, ADAMTS4, ADAMTS5*), as well as inflammatory mediators (*IL6, PTGS2*) that, as well as regulating chondrocyte and synoviocyte metabolism, are associated with pain. The beneficial effects of the derivatized HA did not appear to be simply through blocking IL-1 from reaching cells, as there was no effect on the expected regulation of certain genes by IL-1 e.g. inhibition of *ACAN* in HAC or stimulation of *ADAMTS4* in HSF. The derivatized HA was particularly effective at reducing the IL-1β-induced pro-catabolic and pro-inflammatory gene expression changes. MMP-13 mRNA, protein and activity levels were only reduced by the derivatized HA. Furthermore, the beneficial effects were more pronounced and occurred at a lower dose when the cells (particularly chondrocytes) were pre-incubated with the HA prior to IL-1β exposure. This may suggest that IL-1β and HA are acting via the same signalling pathway, however, HA pre-incubation may also be inhibiting activity of a “secondary” IL-1 induced signalling pathway, for example by blocking an induced collagen peptide binding to ICAM as recently reported [45]. Taken together this data suggests mechanisms whereby the hexadecylamide derivative of HA may have exerted greater inhibition of cartilage degeneration processes, leading to the beneficial effects in OA in our previous in vivo studies [5,19].

The turnover of unmodified HA in the joint is rapid, with a half-life of 27 hours in normal and 11 hours in inflamed ovine joints [46]. The hexadecylamide derivative of HA is an non-chemically crosslinked hydrogel [47] with the capability of completely recovering its viscoelastic properties after several cycles of mechanical stress [48] as well as having a prolonged joint residence time after injection (Fidia SpA, Patent US 7863256). The increased effect with pre-incubation in the current study may suggest this derivative of HA acts not only to ameliorate existing pathology in cartilage and synovium, but could, through its prolonged viscoelasticity and intra-articular retention, prevent the degradative and inflammatory changes with future insults. Resistance to breakdown of the derivatized HA could in part explain its superior effect compared with unmodified HA, where only a limited beneficial effect on gene expression in HAC or HSF ± IL-1β was observed in the current study. The changes in gene expression induced by the derivatized HA did not appear to be associated with altered cell viability as evidenced by the lack of change in cell number, total RNA yield (data not shown), and *GAPDH* mRNA in either cell type, as well as it actually stimulating increased expression of *COL2A1* and *ACAN* in HAC.

Previous studies have reported significant anti-catabolic effects of different unmodified HA preparations in cell and tissue culture [7,16,38,42,43,49,50]. The more limited effect of unmodified HA in the current study could be associated with differences between the unmodified HA preparations evaluated, but also in cell type (human OA, human RA, chondrosarcoma cell lines), dose of HA used (e.g. in some publications effects seen only with >2 mg/ml), and pre-incubation prior to IL-1 stimulation variably used in previous studies. We observed marked differences between patients in basal and stimulated gene expression, as well as the effect of both HA preparations. This could be due to innate variability in patient demographics (e.g. age, disease duration, disease severity) or experimental variability (e.g. topographic differences in sample collection, adaptability of cells to culture and passage number). It is plausible that the differences between patients could reflect the variability observed in those that respond or not to a given treatment, including intra-articular HA. Whether this reflects the difference in initial levels of expression of specific genes could not be examined adequately with this small subset of patients. Nevertheless, the direct comparison between unmodified and derivatized HA in the same patient cells and paired statistical analysis in the current study enabled us to demonstrate a superior effect of the latter formulation.

There is evidence that the mechanism by which IL-1β stimulates MMP production in articular chondrocytes involves phosphorylation of JNK, p38 MAPK and NFκB [51,52]. Previous studies have shown that HA (800 kDa and higher) suppressed IL-1β-stimulated production of collagenases (MMP-1 and MMP-13) by human articular cartilage explants [15] and isolated chondrocytes [38,42], as well as a human chondrosarcoma cell line [53], through down-regulation of phospho-p38 MAPK. In the current study, pre-incubation of HAC with the hexadecylamide derivative of HA not only inhibited p38 phosphorylation but that of NFκB and JNK as well, suggesting all three pathways may be important in IL-1β-stimulated effects in chondrocytes. Inhibition of NFκB by HA has been shown to reduce the production of NO induced by other factors such as fibronectin fragments in chondrocytes [9]. While pre-incubation with derivatized HA ameliorated JNK, p38 MAPK and NFκB phosphorylation in IL-1 stimulated HAC, simultaneous addition did not and there was little or no effect with simultaneous or pre-incubation in HSF, despite significant effects on expression of numerous genes in these cultures. This suggests the importance of alternate pathways for the derivatized HA in regulating gene expression in these cells.

In the present study, the derivatized but not unmodified HA inhibited IL-1-induced *MMP13* mRNA expression in both HAC and HSF. This mRNA regulation was reflected in the MMP-13 activity measured in the HAC but not HSF conditioned media. Furthermore, MMP-13 protein in the media detected by Western blotting was only substantially decreased with the highest dose of derivatized HA. These apparent discrepancies likely reflect the complex regulation of metalloproteinase (MMP and ADAMTS) activity, whereby transcription, matrix and cell-surface binding/sequestration, enzyme activation, inhibitor levels, and enzyme degradation may be differentially affected by the derivatized HA.

It is unclear whether the enhanced suppression of IL-1β-induced gene expression changes by the derivatized compared with unmodified HA, and with its pre-incubation compared with simultaneous addition, are due to direct action on IL-1β signalling or secondary to the enhanced inhibition of *IL6* expression. In both HAC and HSF the changes in expression of *MMP13* and *ADAMTS5* with addition of HA ± IL-1β, largely mirror those of *IL6*. IL-6 alone has been shown to stimulate increased ADAMTS and MMP expression and activity resulting in cartilage degradation [54,55]. Furthermore, IL-6 is a downstream mediator for the effects of IL-1β on chondrocyte collagen synthesis [56], and we observed enhanced *COL2A1* expression by HAC when the derivatized HA was added simultaneously to cultures ± IL-1β. However, despite the even greater reduction in *IL6* expression after pre-incubation with the derivatized HA under these conditions, *COL2A1* expression was not significantly altered. The regulation of *COL2A1* expression by IL-1β in chondrocytes involves an increase in the ratio of Sp1 and Sp3 transcription factors that bind to the *COL2A1* promoter region [57]. In contrast, regulation of catabolic genes (e.g. MMPs, ADAMTS) by IL-1β is largely dependent on NFκB signalling. It is possible that after pre-incubation, the amide derivative of HA is still able to regulate NFκB but does not affect the Sp1/Sp3 levels and this would be interesting to examine in future studies.

It is not clear why the effect of pre-incubation with the amide derivative of HA on IL-1β-stimulated expression was greater in HAC than HSF. HAC and HSF did have a distinct response to IL-1β, as seen with the differential upregulation of *ADAMTS4* and *ADAMTS5* in the two cell types. Furthermore, the response of HSF to IL-1β was approximately 10× greater than HAC with respect to fold increase in MMP expression. Therefore it may be that higher doses of HA or longer pre-incubation may be required for complete inhibition in HSF. As HSF are exposed to greater concentrations of HA within the joint than HAC, they may be less susceptible to exogenous HA-induced metabolic change, perhaps by having a different complement of HA receptors and signalling mechanisms.

CD44 is a multivariate transmembrane glycoprotein known to be the major cell surface receptor for HA, and HA/CD44 interaction plays a central role for the protective effects of HA in various cell types [58-60]. CD44 is abundantly present in the synovium [61] and synovial fluid [62] of the joints of patients with OA, and levels correlate with the degree of inflammation, although not with the degeneration state of the articular cartilage [62]. The interaction of unmodified HA with CD44 is important in suppression of IL-1β-stimulated effects in cultured chondrocytes [15,38,42,53] and synovial fibroblasts [49]. CD44 is also involved in the stimulation of OA chondrocyte proliferation by the amide derivative of HA [22]. We were able to demonstrate amelioration of a few of the effects of the derivatized HA on gene expression using a CD44 blocking antibody in confluent cultures of HAC and HSF supporting a role for this cell-surface receptor. However regulation of expression of most genes by the derivatized HA, particularly in the presence of IL-1 was not altered by blocking CD44 in the current studies. Expression of *CD44* was significantly decreased by derivatized HA in both HAC and HSF (Additional file 4: Figure S3), which may contribute to the limited effect of blocking this cell surface receptor. Taken together this may suggest the importance of alternate pathways, such as via ICAM [45,63], for the derivatized HA in regulating IL-1-induced gene expression changes in these cells.

## Conclusions

The present studies have demonstrated several potential key mechanisms whereby the intra-articular injection of a hexadecylamide derivative of HA may have both symptom and disease-modifying effects in OA. The superior beneficial effects of the derivatized compared with unmodified HA on both chondrocyte and synovial fibroblast expression of catabolic enzymes and inflammatory cytokines/mediators, were consistent with the previously described positive benefits in a large animal model of OA. It remains to be determined whether this hexadecylamide derivative of HA will be superior to other unmodified preparations of HA in clinical trials.

## Competing interests

CBL and MMS received funding to undertake this research through a contract between Northern Sydney Central Coast Health and Fidia Farmaceutici. AS is employed by Fidia Farmaceutici, Abano Terme, Italy. The sponsor was involved in the original study design, but did not participate in the collection or analysis of the experimental data.

## Authors’ contributions

MMS was involved in study design, data collection, assisted in the statistical analysis and drafted the manuscript. AKR performed all of the experimental analysis, data collection and assisted in the statistical analysis. AS participated in study design and final manuscript revision. CBL was involved in study design, interpretation of the data and critical review of the manuscript. All authors read and approved the final manuscript.

## Supplementary Material

Additional file 1: Table S1 Real time PCR primers to human genes used in this study. Click here for file

Additional file 2: Figure S1 HA dose response of HAC gene expression. Dose response of unmodified HA (white markers) and the hexadecylamide derivative of HA (black markers) on HAC expression of the indicated genes in the presence (solid line) and absence (dashed line) of IL-1β (2 ng/mL). Values are mean log fold-change from control (no IL-1 no HA; at zero) from five separate patients. *P* < 0.05* different from no IL-1, no HA control; # different from IL-1, no HA control; ‡ different between the amide derivative and unmodified HA at the same concentration. Click here for file

Additional file 3: Figure S2 HA dose response of HSF gene expression. Dose response of unmodified HA (white markers) and the hexadecylamide derivative of HA (black markers) on HSF expression of the indicated genes in the presence (solid line) and absence (dashed line) of IL-1β (2 ng/mL). Values are mean log fold-change from control (no IL-1 no HA; at zero) from five separate patients. *P* < 0.05* different from no IL-1, no HA control; # different from IL-1, no HA control; ‡ different between the amide derivative and unmodified HA at the same concentration. Click here for file

Additional file 4: Figure S3 Effect of pre-incubation on the HA dose response of HAC gene expression. Dose response on expression of the indicated genes of the hexadecylamide derivative of HA added simultaneously with (black markers, dashed line) or 1 hour before (white markers, solid line) the addition of IL-1β (2 ng/mL) in cultures of HAC. *P* < 0.05 for differences from cultures with IL-1β alone (no added HA; #) or differences +/− pre-incubation (§). Values are mean log fold-change from control (no IL-1 no HA; black dot) from five separate patients. Click here for file

Additional file 5: Figure S4 Effect of pre-incubation on the HA dose response of HSF gene expression. Dose response on expression of the indicated genes of the amide derivative of HA added simultaneously with (black markers, dashed line) or 1 hour before (white markers, solid line) the addition of IL-1β (2 ng/mL) in cultures of HSF. *P* < 0.05 for differences from cultures with IL-1β alone (no added HA; #) or differences +/− pre-incubation (§). Values are mean log fold-change from control (no IL-1 no HA; black dot) from five separate patients. Click here for file
