# Supplementary material for: A hexadecylamide derivative of hyaluronan (HYMOVIS®) has superior beneficial effects on human osteoarthritic chondrocytes and synoviocytes than unmodified hyaluronan
Source: J Inflamm (Lond). 2013 Jul 27;10:26. doi: 10.1186/1476-9255-10-26 (PMC3727958; doi:10.1186/1476-9255-10-26)
Supplement: Additional file 1: Table S1 — Real time PCR primers to human genes used in this study. [file 1476-9255-10-26-S1.doc]

## Supplementary Table 1 - Real time PCR primers to human genes used in this study

| Protein name | Gene name | Accession # | Sequence 5’ to 3’ | T °C | Product (bp) |
| --- | --- | --- | --- | --- | --- |
| Aggrecanase 1 (ADAMTS4) | *ADAMTS4* | AF148213 | F – AGA CAC AGG CAG GGA GAG ACA AAG  R – GGA GAA AAC TTA GTC CTT GGG CTT G | 57 | 110 |
| Aggrecanase 2 (ADAMTS5) | *ADAMTS5* | NM_007038 | F – AAC TCC CAG GAC AGA CCT ACG ATG  R – GCA GAT TCT CCC CTT TCC ACA AG | 59 | 191 |
| Aggrecan* | *ACAN* | NM_001135 | F – TCA CCA TCC CCT GCT ATT TCA TC  R – TCT CCT TGG ACA CAC GGC TC | 56 | 105 |
| CD44 | *CD44* | AY101193 | F – AAA GGA GCA GCA CTT CAG GA  R – TGT GTC TTG GTC TCT GGT AGC | 55 | 128 |
| Collagen type I** | *COL1A1* | BC036531 | F – ACA GGG CGA CAG AGG CAT AAA G  R – AAC AGG ACC AGC ATC ACC AGT G | 60 | 229 |
| Collagen type II* | *COL2A1* | NM_033150 | F – CAG TTC GGA CTT TTC TCC CCT C  R – AGT TTC CTG CCT CTG CCT TGA C | 55 | 129 |
| GAPDH | *GAPDH* | NM_002046.3 | F – TCT CCT CTG ACT TCA ACA GCG AC  R – TCT CTC TTC CTC TTG TGC TCT TGC | 57 | 210 |
| Prostaglandin endoperoxide synthase 2 (COX2) | *PTGS2* | M90100 | F – GAC AGT CCA CCA ACT TAC AAT GCT G  R – GCT GCT TTT TAC CTT TGA CAC CC | 55 | 139 |
| Fibronectin | *FN1* | BC143763 | F – CCA CCA AGA AGT GAC TCG CTT TG  R – GCT ACT GGC TGT GAT TTC GGT C | 56 | 142 |
| a-5-integrin | *ITGA5* | NM_002205 | F – TTC AGT GCC GAG TTC ACC AAG  R – TCA CCA ACA GCC ACA GAG TAT CC | 57 | 215 |
| Interleukin-6 (IL-6) | *IL6* | BC015511 | F – GAA GAT TCC AAA GAT GTA GCC GC  R – GAA GGT TCA GGT TGT TTT CTG CC | 55 | 178 |
| Matrix metalloproteinase-1 (MMP1) | *MMP1* | NM_002421 | F – TGG ACC TGG AGG AAA TCT TGC  R – TCA CAC GCT TTT GGG GTT TG | 55 | 312 |
| Matrix metalloproteinase-13 (MMP13) | *MMP13* | NM_002427 | F – AAA ACG CCA GAC AAA TGT GAC C  R – GCA TCA ATA CGG TTG GGA AGT TC | 55 | 173 |
| Tissue inhibitor of MMPs 1 (TIMP1) | *TIMP1* | NM_003254 | F – GAC ACC AGA AGT CAA CCA GAC C  R – GGT AGT GAT GTG CAA GAG TCC A | 55 | 220 |
| Tissue inhibitor of MMP 3 (TIMP3) | *TIMP3* | NM_000362 | F – TGC CCT TCT CCT CCA ATA CA  R – CTT CCT TCC CTC CCT CAC TC | 55 | 197 |
| Tumor necrosis factor alpha (TNF-a) | *TNF* | NM_000594 | F – CCA ATC CCT TTA TTA CCC CCT CC  R – TGG TTG CCA GCA CTT CAC TGT G | 56 | 171 |

* in chondrocytes only; ** in fibroblasts only
