# Supplementary figures and images for: A hexadecylamide derivative of hyaluronan (HYMOVIS®) has superior beneficial effects on human osteoarthritic chondrocytes and synoviocytes than unmodified hyaluronan
Source: J Inflamm (Lond). 2013 Jul 27;10:26. doi: 10.1186/1476-9255-10-26 (PMC3727958; doi:10.1186/1476-9255-10-26)

## Slide 1
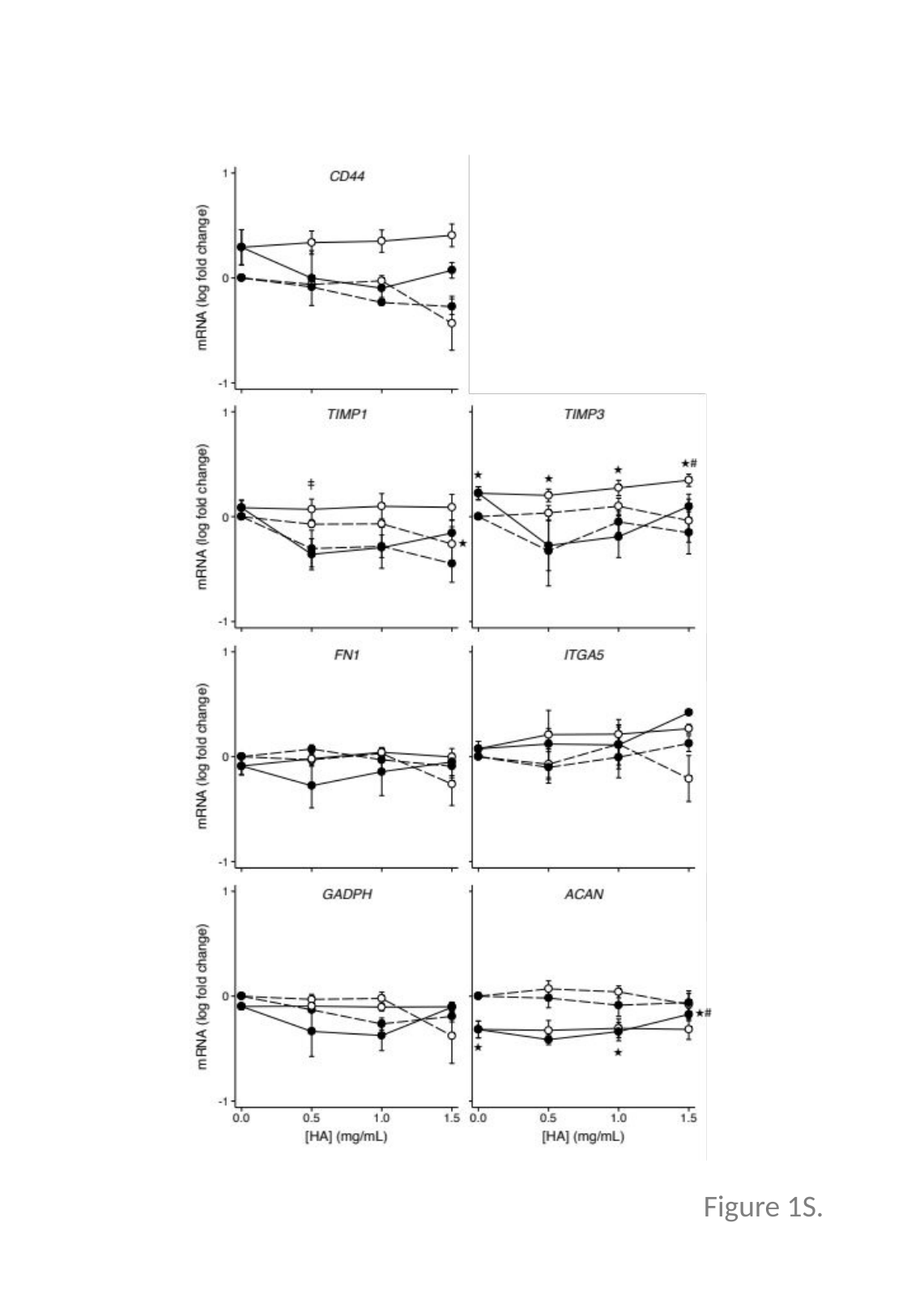

Figure 1S.

Supplement: Additional file 2: Figure S1 — HA dose response of HAC gene expression. Dose response of unmodified HA (white markers) and the hexadecylamide derivative of HA (black markers) on HAC expression of the indicated genes in the presence (solid line) and absence (dashed line) of IL-1β (2 ng/mL). Values are mean log fold-change from control (no IL-1 no HA; at zero) from five separate patients. P < 0.05* different from no IL-1, no HA control; # different from IL-1, no HA control; ‡ different between the amide derivative and unmodified HA at the same concentration. [file 1476-9255-10-26-S2.ppt]

## Slide 1
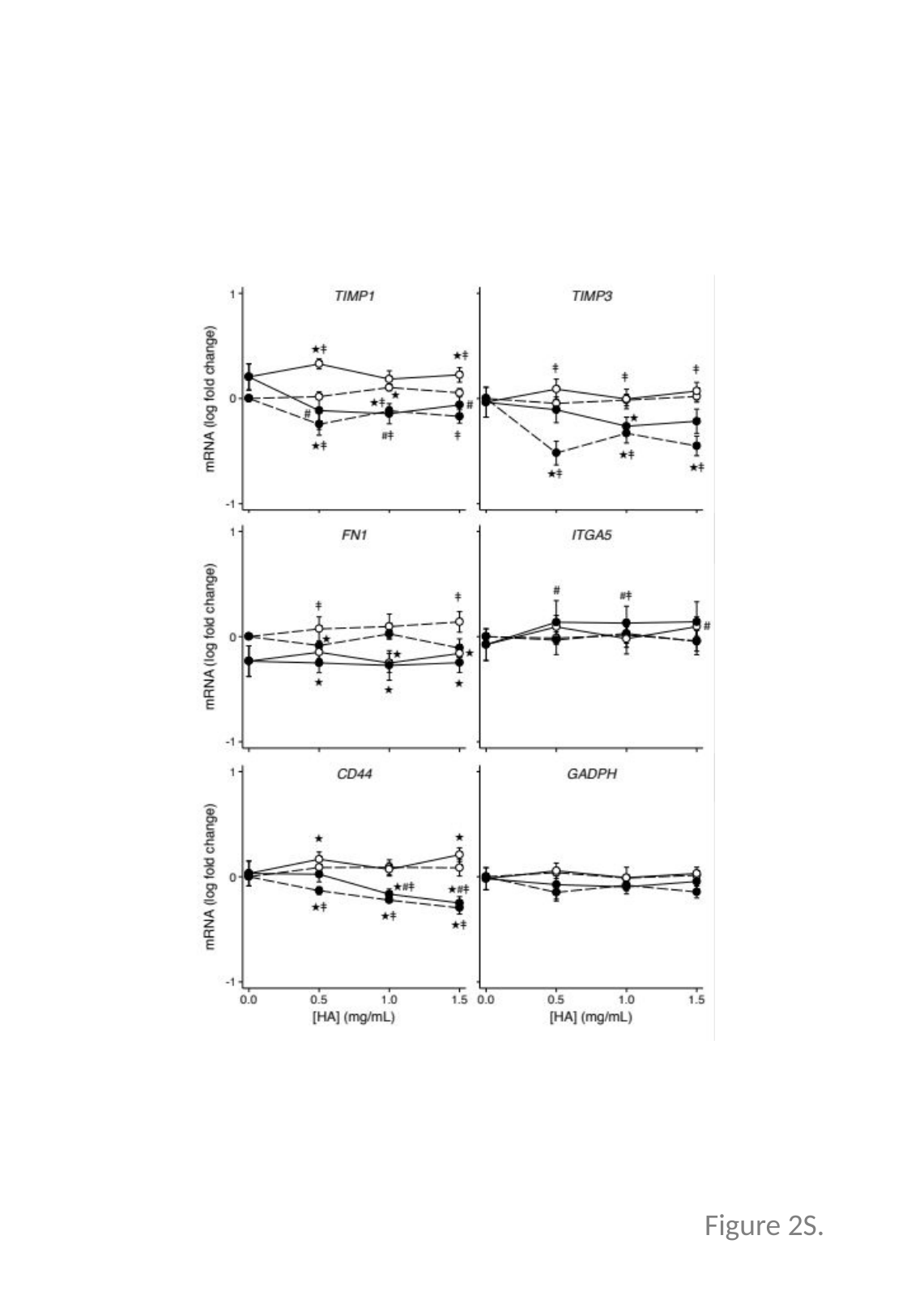

Figure 2S.

Supplement: Additional file 3: Figure S2 — HA dose response of HSF gene expression. Dose response of unmodified HA (white markers) and the hexadecylamide derivative of HA (black markers) on HSF expression of the indicated genes in the presence (solid line) and absence (dashed line) of IL-1β (2 ng/mL). Values are mean log fold-change from control (no IL-1 no HA; at zero) from five separate patients. P < 0.05* different from no IL-1, no HA control; # different from IL-1, no HA control; ‡ different between the amide derivative and unmodified HA at the same concentration. [file 1476-9255-10-26-S3.ppt]

## Slide 1
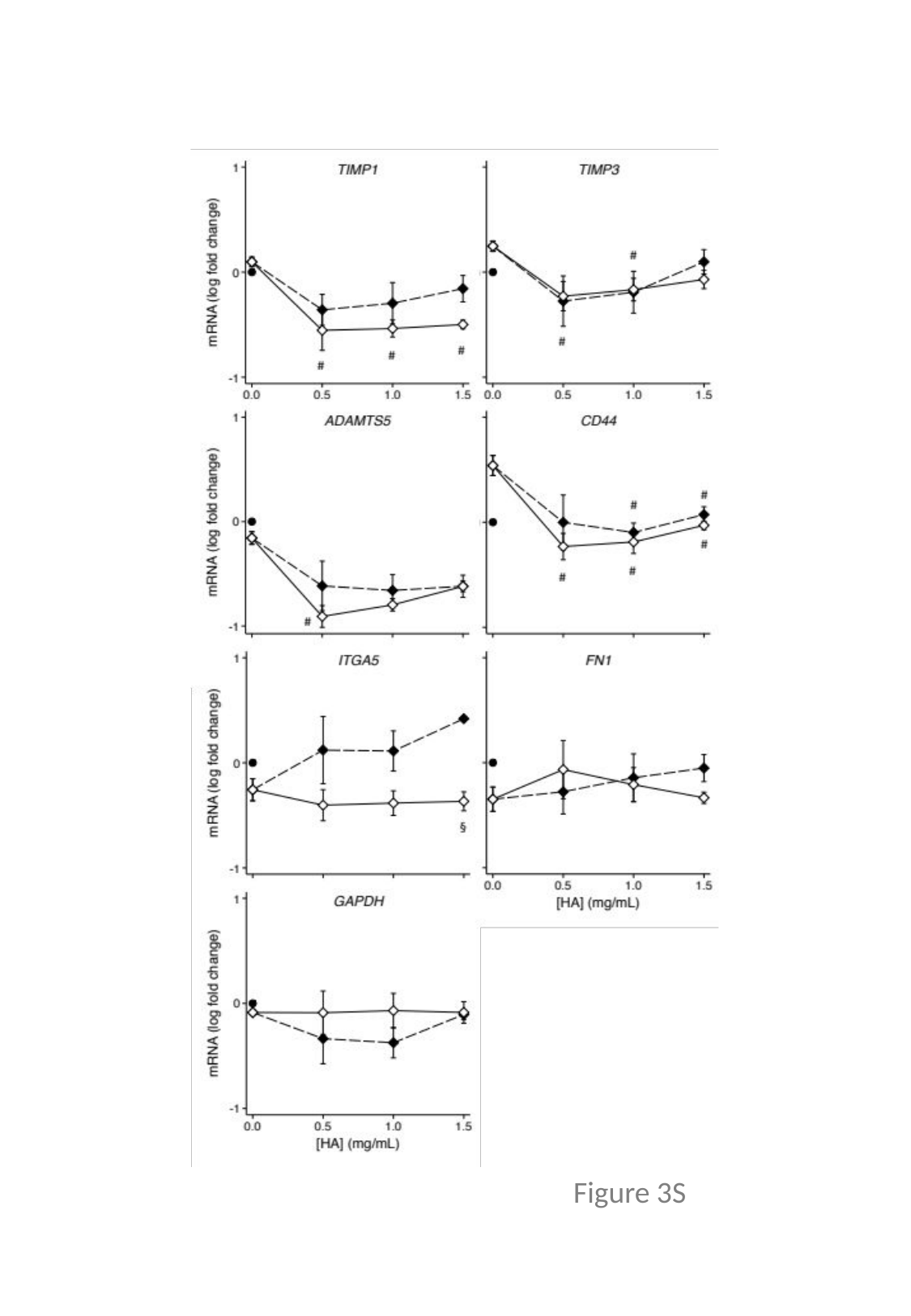

Figure 3S

Supplement: Additional file 4: Figure S3 — Effect of pre-incubation on the HA dose response of HAC gene expression. Dose response on expression of the indicated genes of the hexadecylamide derivative of HA added simultaneously with (black markers, dashed line) or 1 hour before (white markers, solid line) the addition of IL-1β (2 ng/mL) in cultures of HAC. P < 0.05 for differences from cultures with IL-1β alone (no added HA; #) or differences +/− pre-incubation (§). Values are mean log fold-change from control (no IL-1 no HA; black dot) from five separate patients. [file 1476-9255-10-26-S4.ppt]

## Slide 1
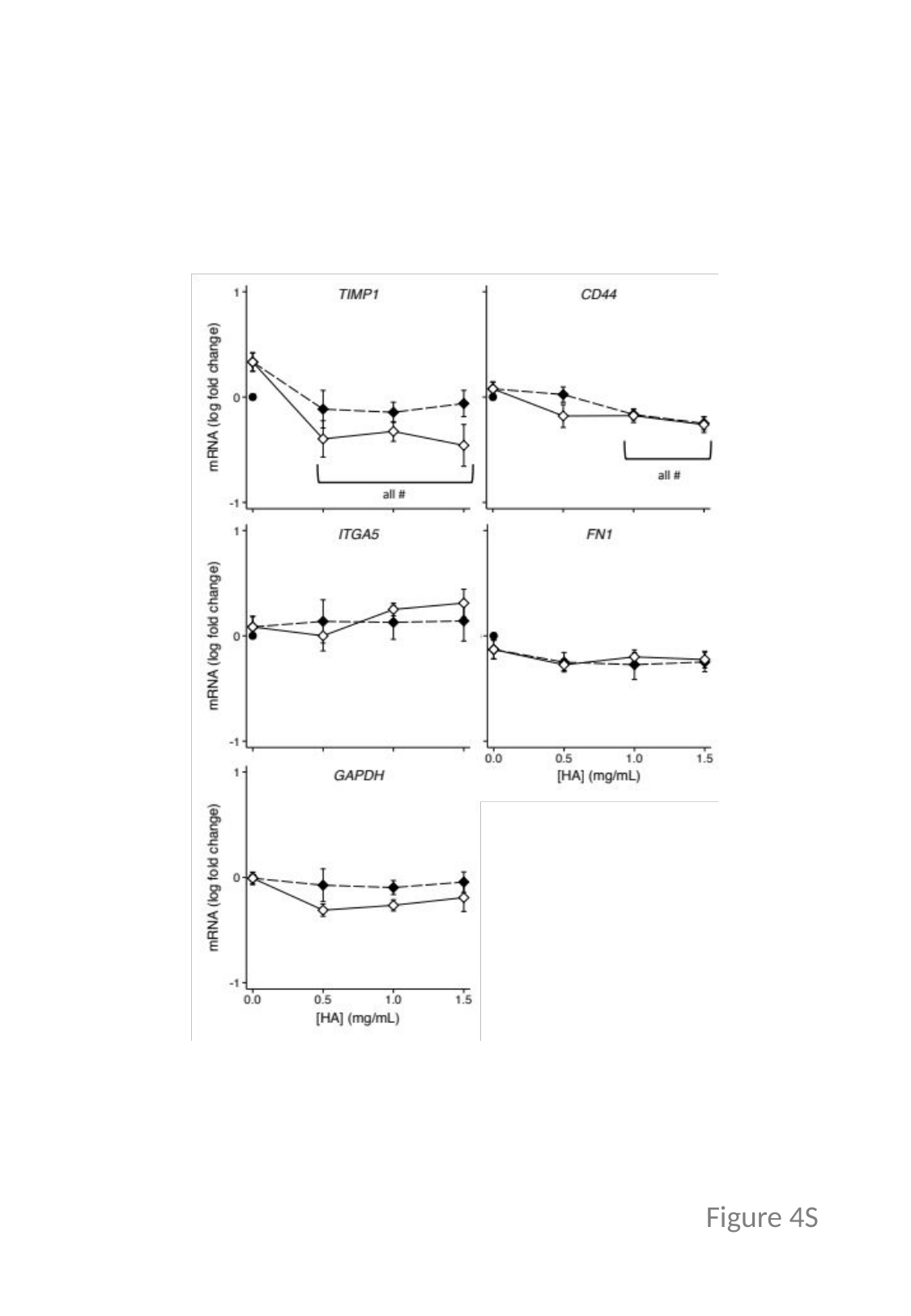

Figure 4S

Supplement: Additional file 5: Figure S4 — Effect of pre-incubation on the HA dose response of HSF gene expression. Dose response on expression of the indicated genes of the amide derivative of HA added simultaneously with (black markers, dashed line) or 1 hour before (white markers, solid line) the addition of IL-1β (2 ng/mL) in cultures of HSF. P < 0.05 for differences from cultures with IL-1β alone (no added HA; #) or differences +/− pre-incubation (§). Values are mean log fold-change from control (no IL-1 no HA; black dot) from five separate patients. [file 1476-9255-10-26-S5.ppt]
